# Mental Stress Detection Using a Wearable In-Ear Plethysmography

**DOI:** 10.3390/bios13030397

**Published:** 2023-03-17

**Authors:** Hika Barki, Wan-Young Chung

**Affiliations:** 1 Department of AI Convergence, Pukyong National University, Busan 48513, Republic of Korea; daljuhika@pukyong.ac.kr; 2 Department of Electronic Engineering, Pukyong National University, Busan 48513, Republic of Korea

**Keywords:** mental stress, scalograms, continuous wavelet transform (CWT), photoplethysmography (PPG), convolutional neural network (CNN)

## Abstract

This study presents an ear-mounted photoplethysmography (PPG) system that is designed to detect mental stress. Mental stress is a prevalent condition that can negatively impact an individual’s health and well-being. Early detection and treatment of mental stress are crucial for preventing related illnesses and maintaining overall wellness. The study used data from 14 participants that were collected in a controlled environment. The participants were subjected to stress-inducing tasks such as the Stroop color–word test and mathematical calculations. The raw PPG signal was then preprocessed and transformed into scalograms using continuous wavelet transform (CWT). A convolutional neural network classifier was then used to classify the transformed signals as stressed or non-stressed. The results of the study show that the PPG system achieved high levels of accuracy (92.04%) and F1-score (90.8%). Furthermore, by adding white Gaussian noise to the raw PPG signals, the results were improved even more, with an accuracy of 96.02% and an F1-score of 95.24%. The proposed ear-mounted device shows great promise as a reliable tool for the early detection and treatment of mental stress, potentially revolutionizing the field of mental health and well-being.

## 1. Introduction

Stress is a common and increasing issue in modern society that can have negative impacts on physical and mental health [1,2]. In particular, mental stress is a major public health issue, with increasing evidence linking it to cardiovascular disease, anxiety, and depression [3]. There is a significant amount of research focused on identifying stress due to the growing evidence linking stress-related health issues to the fast-paced and stressful nature of modern living [4]. Modern stress is in large part the result of pressures at work. Workplace stress can lead to both long-term health problems such as heart disease [5] and sudden, catastrophic outcomes such as accidents, injuries, and even fatalities [6,7]. Due to the potentially life-threatening nature of their jobs, firefighters and smoke divers frequently experience significant levels of stress. Thus, it is important to monitor their mental stress to prevent accidents or injuries [8].

Questionnaires and professional consultations are the most common approaches to diagnosing mental health, but these are expensive, time-consuming, and subjective [9,10] The other approach is using biomarkers such as salivary alpha-amylase and cortisol [11,12]. Although the biomarker method is an objective approach for measuring stress, it has drawbacks, such as the inconvenience of measurement and the inability to continuously monitor stress. As a result, researchers have been working to develop compact, portable, and accurate technology for detecting and monitoring mental stress.

The use of wearable sensors has increased significantly in recent years, allowing researchers to develop a system that can monitor mental health and stress, which can help in quickly detecting and managing these conditions. Research on stress measurement mostly leverages signals from a wearable sensor such as an electrocardiogram [13,14,15], galvanic skin response [16,17], motion sensor [18], and electroencephalography [19,20], and even a combination of these [21]. Our present design employs photoplethysmography, a technology that is widely accepted and recognized for its ergonomic benefits. PPG is commonly integrated into smart rings or wristbands to monitor stress levels [22]. As a wearable technology, PPG is both affordable and non-invasive, making it easy to use. Furthermore, various studies have reported that PPG is not harmful and can be safely used in various clinical settings [23,24,25]. These findings suggest that PPG technology can be effectively used to detect and manage stress levels without causing any harm to the subjects.

Previous studies have used PPG sensors to monitor stress signals with these sensors primarily located at the ear [26,27,28] and wrist [29,30,31,32,33,34]. In those studies, heart rate variability (HRV) was the most used health parameter for stress assessment because it is sensitive to variations in psychological and physical well-being and can distinguish between healthy and unhealthy individuals. However, it can be affected by motion artifacts, which refer to any interference in the PPG signal caused by movement or other external factors. Motion artifacts can distort the PPG signal, making it difficult to accurately measure heart rate variability. Thus, it is important to use a wearable device that is properly secured to the body and to minimize unnecessary movement during the measurement period. This study presents an in-ear wearable biosensor with an IoT-connected platform for stress detection, which is convenient for wearing and reduces motion artifacts compared to other body locations [28]. The authors investigated the use of a device to assess mental stress under controlled laboratory conditions. The stress detection method chosen was based on the continuous wavelet transform (CWT), which is known for its ability to provide more specific information about the frequency patterns of heart rate variability. By utilizing this method, it becomes possible to identify patterns that may indicate stress or other physiological states. To classify the transformed PPG signals into stressed or non-stressed categories, a convolutional neural network (CNN) with a fixed architecture was designed. In comparison to methods that rely on many manually proposed features, the proposed approach using a simple CNN structure may be an efficient way to detect stress in various settings, including workplaces, schools, and sports. However, it is worth noting that traditional classifiers for binary classification, such as decision trees [35,36], random forests [37], and support vector machines (SVMs) [38,39], have also been widely used for binary class classification. These classifiers often require a feature engineering step where relevant features are selected or extracted from the data, which can be time-consuming and may require domain expertise. In summary, this paper makes significant contributions to the field of stress detection through the development of a novel in-ear wearable biosensor and the implementation of an efficient stress detection approach using an effective CNN architecture. The main contributions of this paper are as follows.

1. The design and development of an ear-mounted wearable biosensor for the detection of mental stress.
2. Development of a motion artifact reduction method that employs an adaptive recursive least squares (RLS) filter in conjunction with a dynamic reference signal.
3. Transformation of the 1D PPG signals into 2D time–frequency images (scalograms) using CWT and evaluation of the performance of the transformed signals at different signal segments.
4. The design and implementation of an efficient and accurate CNN model for stress detection using the 2D scalogram images of PPG signals obtained from 14 volunteers.

The paper is organized as follows: Section 2 presents the proposed hardware architecture and stress detection data analysis method. In Section 3, the experimental results are demonstrated. Discussion and conclusion are presented in the final section.

## 2. Materials and Methods

This section discusses the design of a smart in-ear wearable biosensor for monitoring mental stress. Next, the experimental setup for data collection is discussed, followed by a detailed description of the proposed system for mental stress detection.

### 2.1. Proposed Hardware Architecture

**Figure 1:**
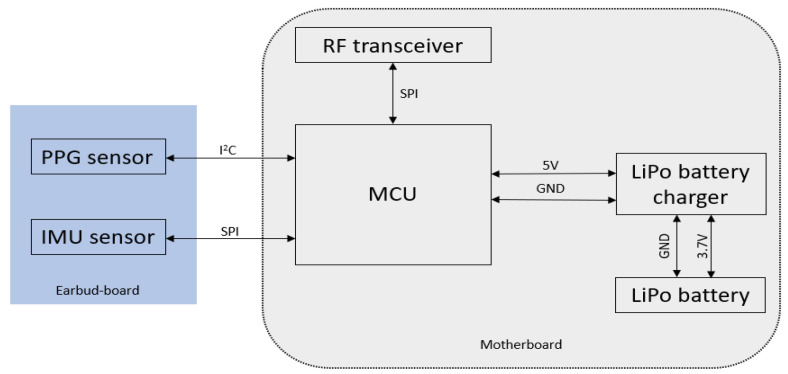
Block diagram of the proposed hardware system.

Figure 1 presents an overview of the proposed system and components used in our design. The system consists of two main modules: (1) a motherboard consisting of a microcontroller unit, power supply, and communication systems; and (2) an earbud board that houses two sensors, a PPG, and an inertial measurement unit sensor for measuring signals from a subject. The following subsections discuss the details of each component that is used in the design of the system.

- Motherboard

The motherboard serves as the central hub of the system, integrating all necessary components such as the microcontroller, communication module, and power supply units. The headband is designed to fit comfortably on the user’s head and securely hold all the necessary components in place. The components housed within the motherboard, including the microcontroller, communication module, and power supply units, will be discussed in further detail in the subsequent sections.

Microcontroller unit: We used the Seeeduino XIAO microcontroller unit (MCU) for our design since it is a small and powerful microcontroller with a wide range of applications. It is manufactured by Seeed Studio (Shenzhen, China), a Chinese company, and is based on the ARM Cortex M4 architecture, known for its high processing power and low power consumption. The microcontroller has 1 MB of flash memory and 128 kB of SRAM, sufficient for many applications. It also has a wide range of peripherals and interfaces, making it easy to connect to other devices and sensors. This microcontroller is packaged in a small 10 × 10 mm^2^ quad flat package, making it easy to integrate into compact systems.

Power supply: Our system was powered by a rechargeable Lithium Polymer (LiPo) battery with a 3.7 V, 500 mAh output, which is known for its high energy density, long cycle life, and stable voltage, making it suitable for portable devices and projects. The Sparkfun Lipo Battery Charger was used to charge this battery; it was designed specifically for LiPo batteries and has several safety features such as power regulation, built-in protection IC, and monitoring charging status and temperature, The charger charges the battery via micro-USB connection to an external power supply, and it also has an on-off button.

Connectivity: The nRF24l01 transceiver module was used for wireless data transmission. It is a wireless communication module manufactured by Nordic Semiconductor, which is a company based in Trondheim, Norway that can be used to transmit data from the device to a central processing system for analysis. It uses the 2.4 GHz industrial, scientific, and medical band and can transmit data over short distances (up to a few hundred meters, depending on the environment). The nRF24l01 module is low-cost and low-power, making it well-suited for use in portable devices such as the stress monitoring system. By integrating the nRF24l01 module into the system, it is possible to transmit data wirelessly from the device to the central processing system for analysis without the need for a physical connection. For this study, the sampling rate for both PPG and IMU signals was set to 100 Hz.

b. - Earbud board

The earbud board consists of two sensors: a photoplethysmography (PPG) sensor and an accelerometer sensor, which allows for measuring vital signs and body movement. These sensors were positioned in the earbud such that they were facing the tragus, a small protuberance on the outer ear. Positioning the sensors toward the tragus can help stabilize the PPG sensor and secure it while it is being worn.

Pulse Oximeter: In our study, we used the MAX30102 reflective pulse oximeter to collect photoplethysmography signals. It is manufactured by Maxim Integrated; a semiconductor company based in San Jose, CA, USA. This sensor is intended for non-invasive measurements of the human body’s oxygen saturation and heart rate, small and low power, making it well-suited for portable and wearable applications. It shines red and infrared LED lights on the tissue and measures the reflected light. The module has features such as optical elements and low-noise electronics to minimize interference from ambient light and improve measurement accuracy. The module can operate with a single power supply of 1.8 V for the digital electronics and another power supply ranging from 3 to 5.25 V for the LEDs. It has an I2C-compatible serial interface and a compact size of 5.6 × 3.3 × 1.55 mm^3^, making it easy to incorporate into small devices.

IMU sensor: A BNO055 module, provided by Bosch Inc., Stuttgart, Germany, is a 9-DOF sensor was used in the study. This module combines a triaxial 14-bit accelerometer, a 16-bit gyroscope, and a triaxial geomagnetic sensor in a single package. In our study, we used only the accelerometer part of the BNO055 module to remove motion artifact from photoplethysmography (PPG) signals. The module’s rapid response time, low noise, and small size made it well-suited for this purpose. By utilizing the triaxial accelerometer, we were able to measure the acceleration of the body part where PPG was being measured, and use this data to remove the motion artifact from PPG signals. Although the full capabilities of the BNO055 module were not used in our study, its advanced design and integration of multiple sensors make it a versatile and powerful sensing device for various applications in robotics, drones, virtual reality, and other motion-sensing devices.

**Figure 2:**
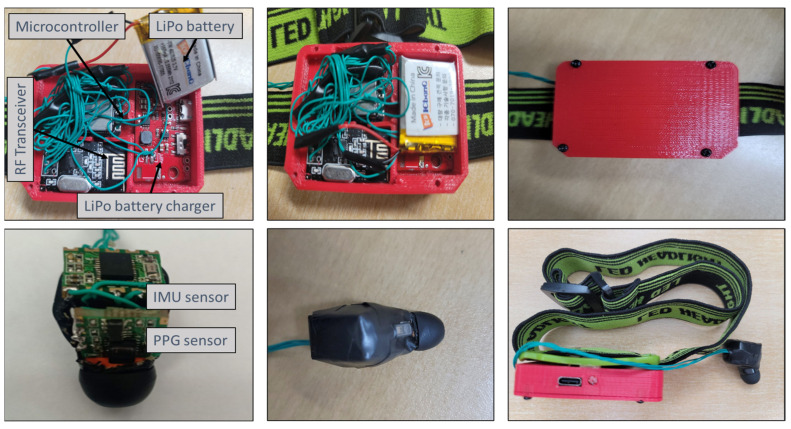
Final design of the motherboard interconnected with the earbud board.

**Figure 3:**
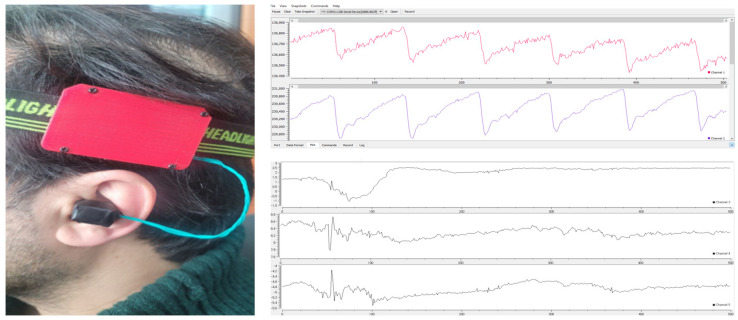
In−ear PPG setup, showing simultaneous recordings of PPG and tri−axis acceleration signals, with accompanying data acquisition software.

In Figure 2, the connections between the components of the system are depicted using wires. Table 1 provides a summary of the components of the sensing module along with their specifications. The motherboard enclosure was designed using 3D modeling software for the purpose of being worn on a headband during data collection (Figure 3). The commercial earbud was modified to include both a PPG and IMU sensor.

### 2.2. Experimental Methodology

**Figure 4:**
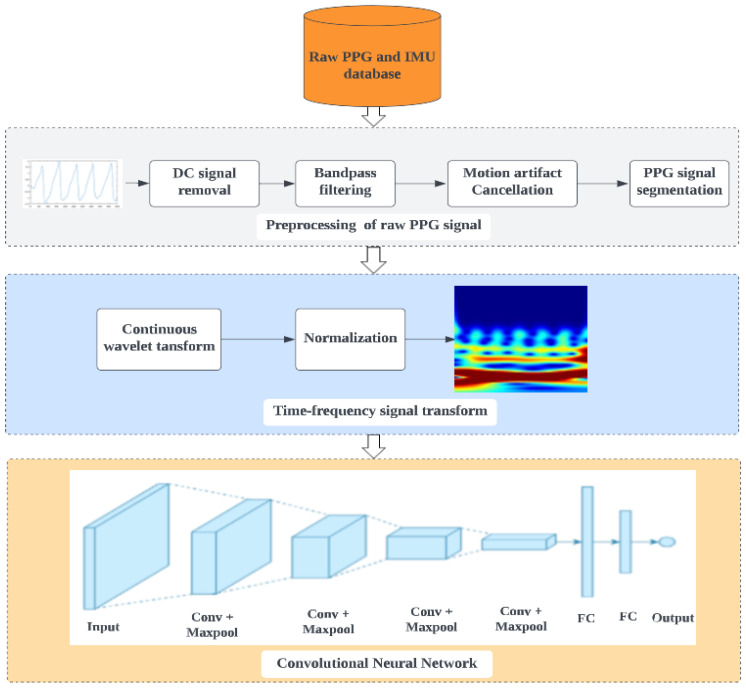
Overall block diagram of the proposed system for mental stress detection.

The experiments in this study were conducted on a workstation computer that had a 64-bit Windows 10 Pro operating system, an AMD Ryzen 5 5600G processor with Radeon Graphics, 16 GB of RAM, and MATLAB version of 2022b. This workstation was chosen because it was powerful and fast enough to handle the demands of the experiments and the proposed stress detection method. The stress detection system consists of four pipelines that work together to detect and measure mental stress in individuals (Figure 4): data acquisition, preprocessing, continuous wavelet transform, and convolutional neural network.

#### 2.2.1. Data Acquisition and Protocol

**Figure 5:**
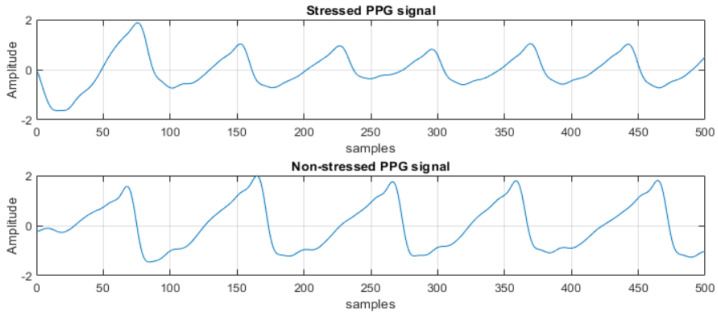
Samples of PPG signals for stressed and non−stressed conditions.

Data for this experiment were collected using the in-ear biosensor in a laboratory at Pukyong National University from 14 healthy volunteers (8 males and 6 females, aged 22–35 years, with BMIs of 17.6–33.8 kg/m^2^). Before the trial, each participant completed a questionnaire about their cardiovascular and mental health and any medications that could potentially affect the results. Participants with hypertension, cardiac arrhythmias, or cognitive impairments were not included in the study. Throughout the trial, the volunteers were subjected to a variety of mental stressors and relaxation activities, including the Stroop color–word test and mental arithmetic tasks. Examples of the collected signals for the stressed and non-stressed conditions are seen in Figure 5. The figure illustrates that a stressed PPG signal may have a lower amplitude and a shorter distance between the two peaks compared to a non-stressed PPG signal. This is because stress can cause changes in the autonomic nervous system, leading to changes in blood flow and vasoconstriction, which can affect the PPG signal.

This study was reviewed and approved by the Pukyong National University’s Institutional Review Board (IRB number: 1041386-202003-HR-13-01). The review process of the IRB included a comprehensive examination of the study protocol and informed consent procedure, as well as an evaluation of potential risks and benefits to participants. Informed consent was obtained from all participants prior to the start of the study. Any deviations from the original study protocol were approved by the IRB.

#### 2.2.2. PPG Signal Preprocessing

**Figure 6:**
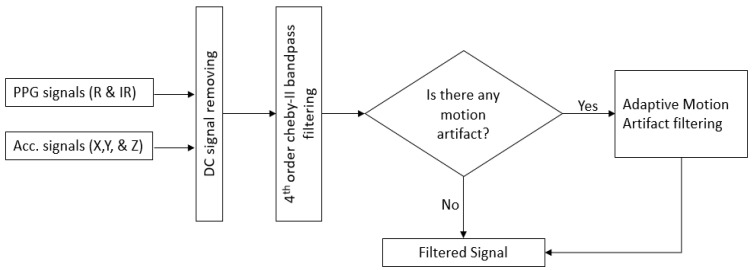
A flowchart of PPG signal preprocessing.

The preprocessing of the raw PPG signal is illustrated in Figure 6. The pipeline includes the input data of PPG and tri-axis accelerometer signals, removal of DC signals, bandpass filtering, and adaptive cancellation of motion artifacts. The following sections provide an explanation of each stage.

- DC Remover

The AC component of the PPG signal is of particular interest in stress detection because it contains the pulsatile information of blood flow that is sensitive to changes induced by stress. Therefore, this study focused solely on the AC component of the PPG signal for stress analysis. To obtain the AC component, we applied the DC remover method, which is an IIR filter design commonly used to remove the low-frequency voltage offset in PPG signals [40]. The following equations explain how the DC remover works.

(1) $$m\left(t\right)=x\left(t\right)+a*m\left(t-1\right)$$

(2) $$y\left(t\right)=m\left(t\right)-m\left(t-1\right)$$

(3) $$\frac{Y\left(Z\right)}{X\left(Z\right)}=\frac{1-Z^{-1}}{1-a*Z^{-1}}$$

where *x(t)* represents the PPG signal’s sampling point input at each time, which is the raw signal obtained from the PPG sensor; *m(t)* represents the calculated value of the operation process, which records the DC drift of the PPG signal; a is the operation parameter that controls the filter cutoff band range and response speed; and *y(t)* represents the AC component of the PPG signal, which is obtained by subtracting the previous value of *m(t)* from the current value of *m(t)*. At a=1, the filter effect is no longer present, but as it approaches 1, the slope of the filter response grows steeper, and only the frequencies that perturb the signal are damped. Equation (3) represents the transfer function of the DC remover filter design, where *X(Z)* and *Y(Z)* represent the z-transforms of the input and output signals, respectively.

b. - Bandpass filtering

There are several forms of noise that might affect the photoplethysmography signal obtained from a photoelectric pulse sensor, such as motion artifacts caused by head movement and random noise during the recording process. Removing these sources of noise will allow for more precise measurements, which can be achieved through bandpass filtering, which may be used to silence noises that are beyond the range of the human pulse (0.5–3.5 Hz). In this study, a 4th-order Chebyshev-II filter was utilized with a bandpass frequency of 0.5–3.5 Hz. Similarly, all three acceleration signals are bandpass filtered to guarantee uniformity. Aside from not utilizing them independently, PPG signals obtained from the infrared and red LED of the Max30102 sensor are normalized and averaged to create a composite PPG signal. As a result, the influence of noise on the signal is reduced, thus making the measurement more precise.

c. - Motion Artifact Cancellation using Adaptive Filtering

Motion artifacts are a persistent challenge for accurate wearable photoplethysmography monitoring, caused by the constant movement of the human body. These can corrupt the waveform of the PPG, leading to improper signal interpretation, and thus these should be minimized. Signal preprocessing techniques must be applied to eliminate any motion artifacts (MAs) present in the recorded signal [41,42].

**Figure 7:**
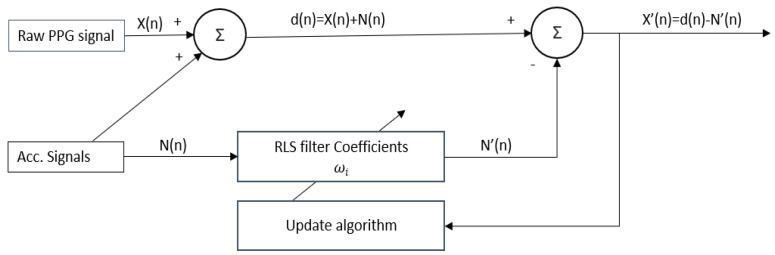
Block diagram of the adopted RLS algorithm.

Many methods have been proposed to reduce the MA of the corrupted PPG signal. These include independent component analysis [43], empirical mode decomposition [44,45,46], Kalman filtering [47], and adaptive noise cancellation (ANC) [48,49,50,51]. Among these techniques, ANC is a well-known and efficient method for reducing MA in a noisy PPG signal. One of the benefits of the ANC is its fast response time, allowing it to quickly adapt to changing conditions. Additionally, ANC can continuously process a signal in real time, even in situations where the noise is rapidly changing. The three most widely used algorithms in adaptive filtering are least mean squares (LMS), normalized LMS (NLMS), and RLS. We adopted an adaptive motion artifact reduction approach based on an RLS adaptive filter since it provides a faster convergence speed and stable filtering. Utilizing the acceleration signal as the reference signal for RLS adaptive filtering to minimize MA is a popular practice nowadays. Nevertheless, the MA removal performance of the adaptive filtering method will be influenced by the selection of a suitable reference signal. Figure 7 illustrates the block diagram of the proposed adaptive RLS filter algorithm.

The effect of motion during data collection may be the combined effect of single or multiple axes. In this method, the tri-axis acceleration components (i.e., X, Y, and Z) were used as the reference input signal of the improved RLS adaptive filter. The accelerometer output is initially preprocessed using the same signal processing methods as a PPG signal by applying the DC remover and bandpass filtering. A stronger correlation between the reference signal and the MAs results in improved motion artifact reduction. The Pearson correlation coefficient (*r*) [52] was used to find a suitable reference signal among the three axes of the accelerometer signal. A higher absolute correlation value indicates greater similarity between the PPG and reference signal. The absolute correlation coefficient (|*r*|) between the PPG and acceleration signal (reference signal) is computed using equation 4. In this study, the maximum absolute correlation coefficient (*|r|_max_*) among the three computed values was used to check if there is a high or low correlation between PPG and acceleration signals. The acceleration signal with the maximum absolute correlation coefficient was used as a reference signal. In our case, we empirically set the threshold at *|r|_max_* > 0.3 to indicate a strong correlation between the PPG and acceleration signals.

(4) $$\left|r\right|=\left|\frac{\sum (PPG_{i}-\bar{PPG})\left(Acc_{i}-\bar{Acc}\right)}{\sqrt{\sum {\left(PPG_{i}-\bar{PPG}\right)}^{2}\sum {\left(PPG_{i}-\bar{PPG}\right)}^{2}}}\right|$$

where $PPG_{i}$ represents the primary signal, $Acc_{i}$ represents the reference acceleration signal, $\bar{PPG}\;$ and $\bar{Acc}$ are the arithmetical mean values of the primary and reference signals, respectively.

In the RLS algorithm, there are two primary inputs: *X(n)* and *N(n). X(n)* represents the PPG signal, which includes MAs, whereas *N(n)* is one of the tri-axis accelerometer signals. As shown in Figure 7, the output signal, *X’(n),* can be obtained by using the following equation:

(5) $$X^{\prime }\left(n\right)=d\left(n\right)-N^{\prime }\left(n\right)$$

(6) $$N^{\prime }\left(n\right)=\omega^{T}\left(n\right)N\left(n\right)$$

From Equations (5) and (6), we can see that the optimal weight vector $\omega^{\;}$(n) should be iterated to obtain the output of the signal. The cost function of the RLS adaptive filtering *J(n)* is provided as in the following equation, where λ is the forgetting factor.

(7) $$J\left(n\right)=\sum_{n=1}^{k}\lambda^{k-n}{\left|X^{\prime }\left(n\right)\right|}^{2}=\sum_{n=1}^{k}\lambda^{k-n}{\left|d\left(n\right)-\omega^{T}\left(n\right)N\left(n\right)\right|}^{2}$$

Taking the derivative of the weight vector $\;\omega^{\;}\left(n\right)\;$ yields the minimum value of the cost function $J\left(n\right)$. This can be written as:

(8) $$\omega_{opt}\left(n\right)=R^{-1}\left(n\right)*r\left(n\right)$$

where $R\left(n\right)=\sum_{n=0}^{k}\lambda^{k-n}u\left(n\right)u^{T}{\left(n\right)}^{\;}$ and $r\left(n\right)=\sum_{n=0}^{k}\lambda^{k-n}u\left(n\right)d{\left(n\right)}^{\;}$. Using the above equations, we can compute the output signal, $X^{\prime }\left(n\right).$

The forgetting factor, λ, is an adjustable parameter in the RLS algorithm that determines how much prior data the algorithm accounts for. It has a decaying value that decreases over time, which in turn reduces the influence past data points have on the model’s coefficients. When set to 1, the algorithm utilizes all available data for estimating the model’s parameters, not disregarding any information. However, if the data are non-stationary and the trends are changing, using a value of λ less than 1 can enable the RLS algorithm to adapt more swiftly to these changes. The forgetting factor determines the balance between the model’s ability to adapt to new trends and its stability. A higher value leads to more stability but slower adaptation, whereas a lower value results in faster adaptation but may also introduce noise and instability in the model’s parameter estimates. In this study, we used a forgetting factor of λ = 0.999.

**Figure 8:**
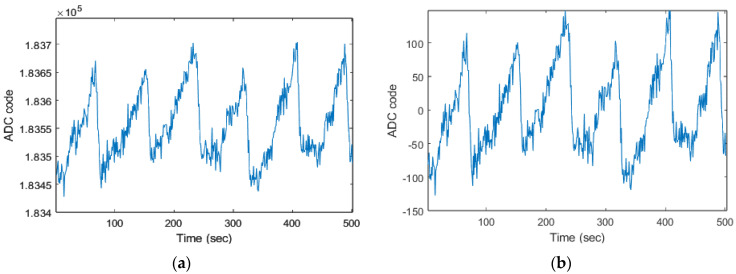

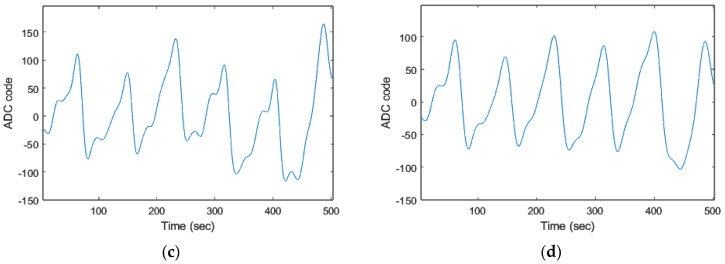
Preprocessing of the raw PPG signal: (**a**) original, (**b**) DC component removed, (**c**) Band−pass filtered, and (**d**) reduced motion artifacts.

Figure 8 illustrates a sample of a raw PPG signal that has undergone various preprocessing steps to prepare them for further analysis. One of these steps is DC signal removal, which is depicted in Figure 8b. This process eliminates the constant offset in the signal that can cause problems with further processing. Another step is bandpass filtering, shown in Figure 8c; this step removes unwanted high-frequency and low-frequency noise from the signal. The last step is motion artifact reduction using an adaptive RLS filter, also shown in Figure 8c; the filter uses an RLS algorithm to adaptively estimate the parameters of the filter and reduce MAs.

d. PPG Signal Segmentation

This experiment aims to classify signals based on their length, specifically comparing the performance of segments lasting 3 and 5 s. Signals that were 3 min long were obtained for each class per subject, and then divided into 2 non-overlapping segments for classification (Table 2). The experiment used 11 subjects for training and 3 subjects for testing. By comparing the performance of the different segment lengths, the experiment aims to determine which segment length is most effective for signal classification.

#### 2.2.3. PPG Signal Transformation

**Figure 9:**
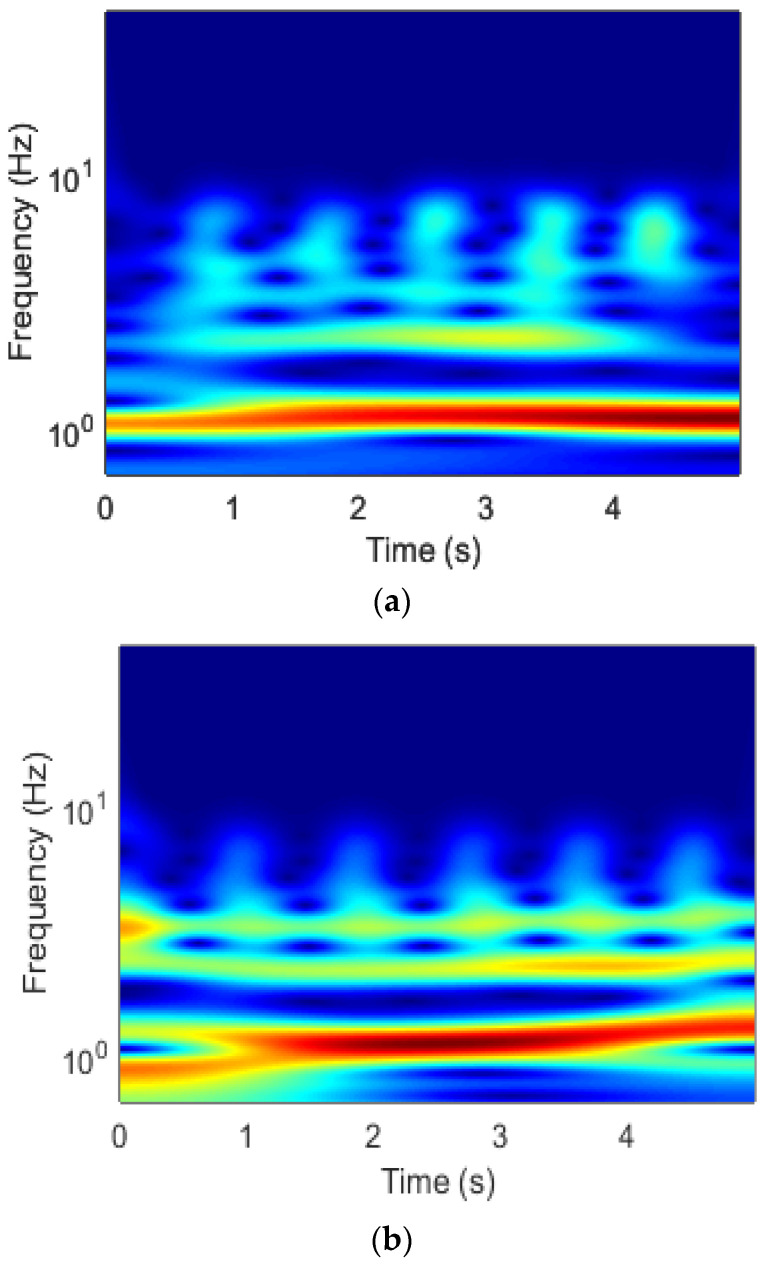

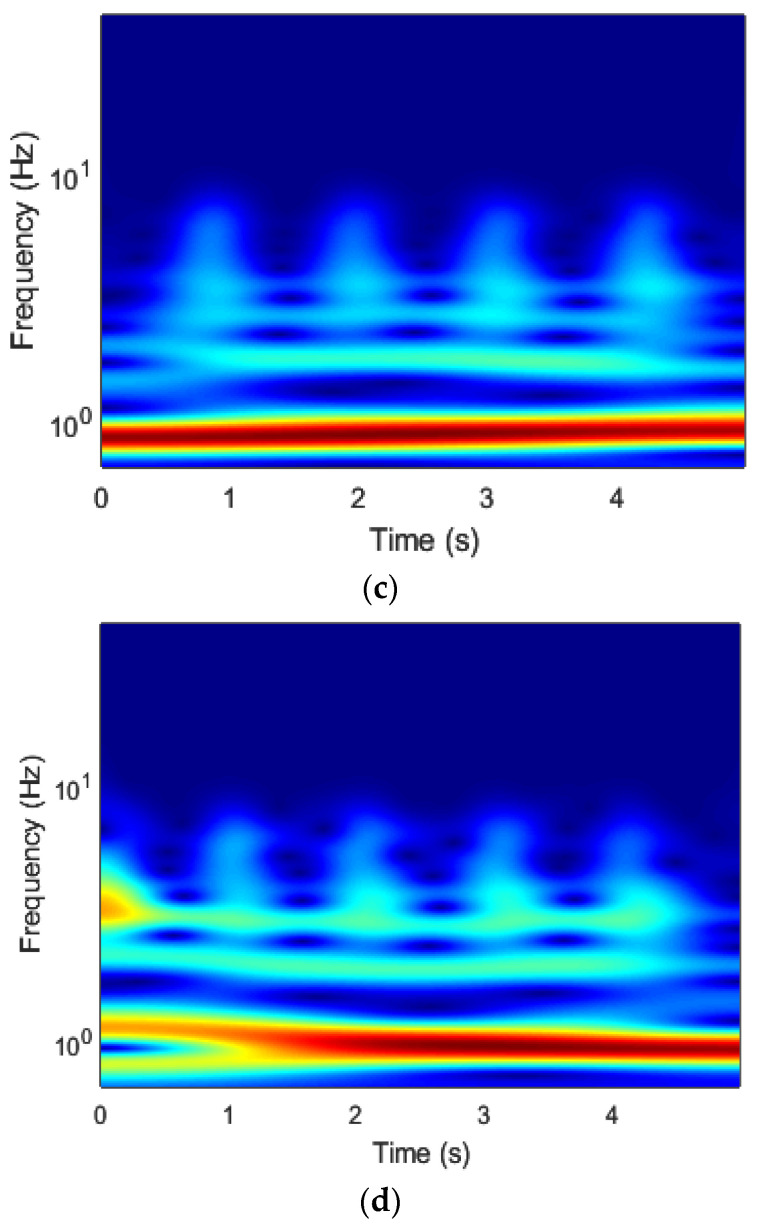
Examples of signals that have been transformed using the generalized Morse wavelet, in both (**a**,**b**) stressed and (**c**,**d**) non-stressed conditions, from various subjects.

The PPG signal comprises many frequency components that correlate to various physiological functions that are helpful in detecting stress. Different signal processing methods, such as the continuous wavelet transform (CWT), may be used to extract and evaluate these frequency components. The CWT is a popular tool for analyzing signals in the time–frequency domain. It works by using a set of wavelet functions to decompose a signal, allowing for the identification of specific frequencies and their corresponding amplitudes at different points in time. This technique has been widely used in a variety of applications, particularly in signal processing and image analysis. The CWT was utilized in this experiment to convert the PPG signal into a time–frequency representation, known as a scalogram, which was then fed into a convolutional neural network (CNN) to identify mental stress. Formally, given a signal *x(t),* the CWT is defined as:

(9) $$C_{x}\left(a,b\right)=\frac{1}{\sqrt{a}}\int_{\infty }^{\infty }x\left(t\right).\omega \left(\frac{t-b}{a}\right)dt$$

where *a* is the scale parameter, *b* is the translation parameter, and $\omega \left(t\right)$ is the wavelet function. The CWT employs scale parameters to give high-time and low-frequency resolution for identifying high-frequency events in the PPG signal, as well as low-time and high-frequency resolution for detecting low-frequency events [53]. Equation (10) demonstrates the frequency-domain representation of the scale parameter.

(10) $$F=\frac{F_{c}*f_{s}}{a}$$

where $F_{c}$ is the center frequency of the mother wavelet, and $f_{s}$ is the sampling frequency of signal *x(t)* [54]. A CWT filter bank is used to create the scalogram images for the stressed and non-stressed conditions. Different wavelet functions, or ‘mother wavelets’, can be used in the CWT to decompose a specific signal into the time–frequency domain. The selection of the wavelet function should be carefully chosen to optimize the decomposition of the signal. In this study, the generalized Morse wavelet was used as the mother wavelet, defined as:

(11) $$\varphi_{\beta ,\gamma }\left(t\right)=\frac{1}{2\pi }\int_{0}^{\infty }a_{\beta ,\gamma }\omega^{\beta }e^{-\omega \gamma }e^{i\omega t}d\omega$$

where $a_{\beta ,\gamma }$ is the normalizing constant, γ characterizes the Morse wavelet’s symmetry, and β is the compactness parameter. The PPG signals were segmented into two sets (3 and 5 s sets) and transformed into 224×224 pixel scalograms for use as input to the proposed CNN. Figure 9 illustrates two scalogram images generated for two different classes of PPG signals using a filter bank.

#### 2.2.4. Proposed CNN for Mental Stress Detection

CNNs are a type of deep learning algorithm that uses convolutional operations to replace general multiplications in traditional neural networks. These are particularly useful for classification tasks, particularly in image recognition, due to their ability to automatically extract discriminatory features through the training process. In recent years, CNNs have been adopted in a variety of applications [55]. In this research, we designed a simple yet effective CNN, which has many typical structures: a convolutional layer, a pooling layer, a fully connected layer, and a dropout layer.

**Figure 10:**
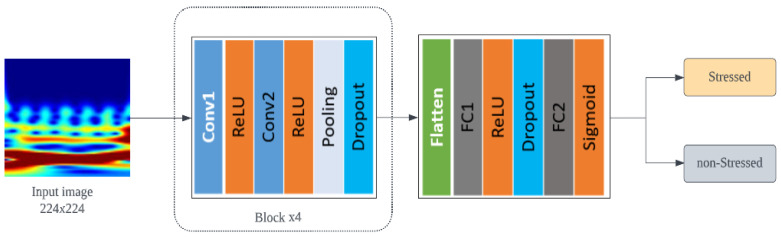
An overview of the proposed 2D-CNN method for mental stress detection.

The proposed architecture is shown in Figure 10. The network is composed of three main parts: an input layer for accepting input data, convolutional blocks for extracting deep features from the input tensor, and a fully connected layer for combining all the features extracted by the convolutional blocks and producing the final classification result. The configurations of each layer of the network are listed in Table 3. *Input layer:* the input to the proposed CNN comprises 3-channel scalogram images 224×224 pixels in size. *Convolution block:* the network consists of four convolutional blocks, each of which includes two convolution layers, a pooling layer, and a dropout layer. The convolution layers use 64 kernels measuring 3×3 pixels with a step size of 2 and the ReLU activation function. Following the processing of the input tensor by the 2 convolutional layers, a maximum pooling of 2×2 pixels is carried out to minimize the number of features. The dropout layer has a rate of 0.25 and is used to prevent overfitting and improve training speed by randomly dropping out some neurons during each training iteration [56]. *Fully connected layer:* the input matrix undergoes four convolutional blocks to extract deep features with multiple channels. These features are then combined and flattened into a single dimension and passed through a fully connected layer with 128 cells and a ReLU activation function. A dropout layer with a dropout rate of 0.5 was added after this layer. The final fully connected layer outputs the classification result, which is a single class (stressed/non-stressed) with a sigmoid activation function representing the probability that the input matrix belongs to a particular category.

The proposed CNN model was optimized by adjusting its hyperparameters, such as using a batch size of 32, a learning rate of 0.0001, and utilizing 3 different optimizers (Adam, RMSprop, and SGDM), as listed in Table 4. Through this fine-tuning process, the model was able to achieve the best results in terms of accuracy and other performance metrics. During the training phase, the binary cross-entropy loss function was employed to measure dissimilarity between the predicted probability distribution and the true distribution. This loss function is commonly used in binary classification problems.

#### 2.2.5. Performance Evaluation

We evaluated the effectiveness of our proposed approach in predicting mental stress using four commonly used metrics: accuracy, precision, recall, and F1-score [57]. Each metric is defined as follows in Table 5, where TP stands for true positive, TN for true negative, FP for false positive, and FN for false negative. These metrics can be used to evaluate the performance of the model in terms of how well it is able to correctly classify mental stress (positive cases) and no stress (negative cases).

## 3. Experimental Results

We evaluated our proposed method using two different signal segments (3 s and 5 s segments), with results detailed in Table 6 and Table 7. The results show that our proposed method achieved high accuracy, precision, recall, and F1-scores for both the 3 s and 5 s signal segments. The Adam optimizer provided the best results for the 3 s segment, while the RMSprop optimizer performed best for the 5 s segment. These findings suggest that the performance of our proposed method is influenced by the signal segment length and the choice of optimizer. Overall, our results demonstrate the potential of our proposed method for accurately classifying signal segments. However, it is important to interpret these results with caution, as further investigation is needed to identify the underlying factors behind the performance differences between the optimizers and to address any potential sources of bias or error.

**Figure 11:**
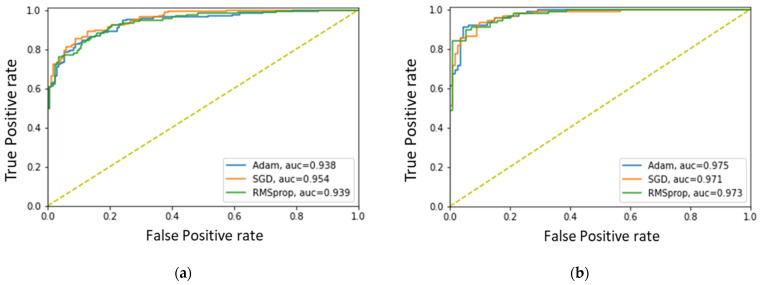
ROC curves of the proposed method using the (**a**) 3 s and (**b**) 5 s signal segments.

Figure 11 illustrates the receiver operating characteristic (ROC) curves of the proposed method for different signal lengths. The classifier was trained using different optimizers and segments of data, and ROC curves were generated to evaluate the performance. The best performance was seen with the SGDM (AUC = 0.954) and Adam (AUC = 0.975) optimizers, for the 3 and 5 s segments, respectively. This suggests that the classifier trained with the Adam optimizer had the greatest accuracy in distinguishing between positive and negative classes. Overall, the Morse wavelet with a 5 s segment and Adam optimizer demonstrated the highest diagnostic accuracy among the various configurations evaluated.

**Figure 12:**
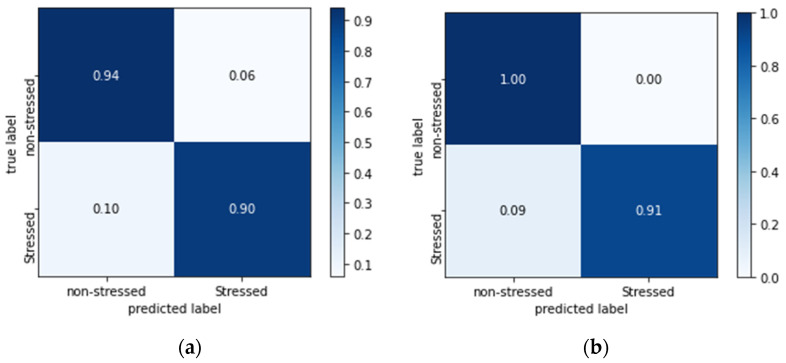
The confusion matrices for the combination of the best-performing signal segment (5 s) and optimizer (RMSprop) (**a**) before and (**b**) after data augmentation.

**Figure 13:**
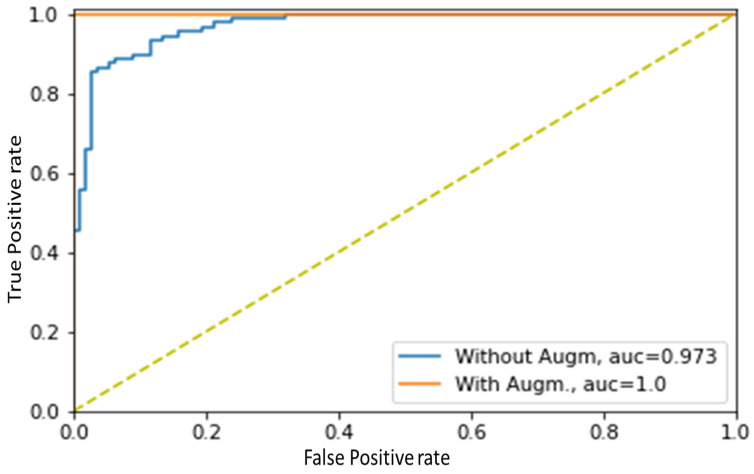
ROC analysis for the combination of the best-performing signal segment (5 s) and optimizer (RMSprop) are shown before and after data augmentation.

To improve performance, we decided to increase the size of the training dataset by adding white Gaussian noise with a mean value equal to 30% of the original scalogram. This noise contamination was included to make the model more robust [58], and the size of the train dataset doubled from 792 to 1584 as a result. The data augmentation significantly increased the accuracy of the experiment from 92.04% to 96.02% (Table 8, Figure 12). This indicates that the model was able to learn more effectively when presented with a larger and more diverse dataset, which contained a greater variety of signal samples. Additionally, the researchers conducted a ROC analysis to evaluate the diagnostic accuracy of the model, which revealed that data augmentation had resulted in an increase in the model’s AUC-ROC value from 0.973 to 1.0 (Figure 13). This suggests that the data augmentation improved the ability of the model to correctly classify positive and negative samples. In short, data augmentation improved the performance of the experiment, resulting in a more robust and accurate model for signal classification.

It has been challenging to compare the results of this study with those of other methods due to the unique nature of the dataset used. We compared our results with those of other state-of-the-art CNN transfer learning methods, including GoogleNet [59], ResNet-50, ResNet-101 [60], and DenseNet-201 (Table 9) [61]. To ensure a fair comparison, all experimental settings were the same as those used in our proposed CNN model. In short, the proposed CNN method was more effective for mental stress detection compared to the CNN-based transfer learning methods. Additionally, our network resulted in shorter training time.

## 4. Discussion and Conclusions

In this research, we proposed a method for detecting mental stress by utilizing an ear-mounted biosensor. The ear-mounted PPG sensor was chosen for its non-invasiveness and convenience, as well as its ability to minimize MAs. Through signal processing and transformation, we were able to classify stressed and non-stressed states using a robust CNN with high accuracy. Our method demonstrated a high classification accuracy of 92.04% in identifying mental stress, as well as a high precision of 93.90%, meaning a high proportion of its positive predictions were accurate. The recall was 87.55%, demonstrating the method’s ability to identify a high proportion of actual positive instances. The F1-score of 90.06% further shows that precision and recall are balanced and high. These results demonstrate the effectiveness of our proposed method in detecting mental stress and its potential for use in monitoring mental stress in various settings, such as occupational health and wellness programs.

This research also examined the impact of signal length on the accuracy of mental stress detection using CWT and CNNs. Using a signal length of 5 s, rather than 3 s, achieved better accuracy in detecting mental stress, likely because longer segments of signals contain more data, which can provide more information for the analysis. However, longer segments may also have more noise and lower frequency resolution. On the other hand, shorter segments may contain less data, but they may have higher frequency resolution. These trade-offs should be considered when selecting the length of the signal for mental stress detection; it is important to strike a balance between having enough data for analysis and having high-frequency resolution to obtain more insights. Additionally, the results of this specific research may not be generalizable to all mental stress detection scenarios, and it would be important to test the results on different data sets and real-life applications.

Moreover, this study demonstrates that the performance of a CNN can be greatly affected by the choice of optimizer. There are several commonly used optimizers, such as SGD, Adam, and RMSprop, each with their own unique characteristics that may make them better suited for certain tasks. It can be useful to experiment with a few different optimizers and compare their performance on a particular task to determine the best one to use. Properly tuning the hyperparameters of the optimizer, such as the learning rate, can also contribute to improved performance. 

Our study has some limitations that should be acknowledged. One limitation is the relatively small sample size, which means that our results may not be generalizable to a larger population. This could limit the ability to draw broad conclusions from our findings. Another limitation is that the subjects in our study were only exposed to a limited range of stressors. This may not fully represent the complexity of mental stress in real-world situations, and therefore, our results may not be fully applicable to other types of stressors. Future research could address these limitations by increasing the sample size and recruiting participants from a more diverse population. Additionally, future studies could expose participants to a wider variety of stressors, to better understand how mental stress is affected by different types of stressors.

In conclusion, the proposed method of utilizing ear-mounted PPG in our study is a promising approach for mental stress detection. The system was able to accurately detect mental stress in real time with a high level of accuracy. The use of PPG sensors, which are non-invasive and easy to wear, makes this approach a convenient and practical solution for mental stress detection. However, further research is needed to confirm these results and explore the full potential of this method. One important aspect of further research would be investigating the potential of the method for continuous monitoring of mental stress in a variety of different situations. This may include exploring the use of this method in different populations, such as in different age groups, genders, or cultural backgrounds. Additionally, it could be useful to test the method in different settings, such as in the workplace, home, or educational environments where mental stress is a concern. Another important aspect of further research is to examine the limitations of the method more closely, in order to identify areas where improvements could be made. For example, the study could look at factors that affect the accuracy of the method, such as the impact of different types of noise, or the effect of MAs on the measurements. Furthermore, the study could also try to identify potential biases in the dataset or potential confounding variables that might have influenced the results. By addressing these limitations, the method could become more effective and widely applicable for detecting mental stress.

## Figures and Tables

**Table 1:** Specifications for the components of the system.

Components	Specification
MAX30101 sensor	Operating Voltage: 1.8 V Operating Temp. (°C): −40 to +85 Size: 5.6 mm × 3.3 mm × 1.55 mm
BNO055 sensor	Acceleration Ranges: ±2 g/±4 g/±8 g/±16 g Operating Voltage: 3–5 V
Seeeduino XIAO MCU	Operating Voltage: 3.3 V/5 V CPU: 40 MHz ARM Cortex-M0+ Flash Memory: 256 Kb RAM: 32 KB Size: 20 mm × 7.5 mm × 3.5 mm I2C: 1 pair
nRF24l01module	Operating Voltage: 1.9–3.6 V Modulation: GFSK Data Rate: 250 kbps, 1 Mbps, and 2 Mbps Size: 2.9 cm × 5 cm × 1.2 cm
LiPo battery	Operating Voltage: 3.7 V Capacity: 500 mAh
LiPo battery charger	Capacity: 5 V, 1 A output

**Table 2:** Number of training and testing data by type of length of signal.

Length of Signal	Number of Training Data	Number of Testing Data
3 s	1320	360
5 s	792	216

**Table 3:** A summary of the setups of each layer of the network in the proposed CNN model.

Layer	Output Number	Kernel Size/Pool Size	Stride	Activation Function	Padding	Dropout Rate
Input layer	1	-	-	-	-	-
Conv1-1	64	3 × 3	2 × 2	ReLU	same	-
Conv1-2	64	3 × 3	2 × 2	ReLU	same	-
Maxpool	64	3 × 3	2 × 2	ReLU	valid	-
Droupout1	64	-	-	-	-	0.25
Flatten	-	-	-	-	-	-
FC1	128	-	-	ReLU	-	-
Droupout2	128	-	-	-	-	0.5
FC2	1	-	-	Sigmoid	-	-

**Table 4:** Hyperparameters setting.

Parameter Tuning for CNN	
Optimizer	Adam, RMSprop, SGDM
Learning Rate	1 × 10^−4^
Max. Epochs	50
Validation Patience	10

**Table 5:** Evaluation metrics.

** $Accuracy=\frac{TP+TN}{TP+FP+FN+TN}$ **
** $Precision=\frac{TP}{\left(TP+FP\right)}$ **
** $Recall=\frac{TP}{\left(TP+FN\right)}$ **
** $F1\;score=\frac{TP}{TP+\frac{1}{2}\left(FP+FN\right)}$ **

**Table 6:** Classification results of the proposed method using 3 s segments.

Optimizers	LR	Accuracy (%)	Precision (%)	Recall (%)	F1-Score (%)	Elapsed Time
Adam	0.0001	86.01	84.39	87.95	86.14	21 min 11 s
RMSprop	0.0001	88.1	85.8	90.96	88.3	23 min 36 s
SGDM	0.0001	86.61	86.67	86.14	86.4	52 min 52 s

**Table 7:** Classification results of the proposed method using 5 s segments.

Optimizers	LR	Accuracy (%)	Precision (%)	Recall (%)	F1-Score (%)	Elapsed Time
Adam	0.0001	91.54	87.91	90.91	89.39	9 min 49 s
RMSprop	0.0001	92.04	91.86	89.77	90.8	14 min 38 s
SGDM	0.0001	90.05	94.74	81.82	87.8	28 min 55 s

**Table 8:** Comparison of the best performing method with and without data augmentation.

Evaluation Metrics (in %)	Without Data Augmentation	With Data Augmentation
Accuracy	92.04	96.02
Precision	91.86	100
Recall	89.77	90.91
F1-Score	90.8	95.24
AUC	97.3	100
Elapsed time	14 min 38 s	36 min 35 s

**Table 9:** Comparative analysis with existing CNN models.

Models	LR	Optimizer	Accuracy	Precision	Recall	F1-Score	Elapsed Time
GoogleNet	0.0001	RMSprop	90.1	93.54	86.13	89.69	21 min 11 s
ResNet101	0.0001	RMSprop	90.59	89.22	89.11	90.58	32 min 56 s
ResNet-50	0.0001	RMSprop	90.59	92.71	88.12	90.56	35 min 43 s
DenseNet-201	0.0001	RMSprop	88.61	86.79	91.09	88.89	33 min 46 s
Proposed CNN	0.0001	RMSprop	92.04	91.86	89.77	90.8	14 min 38 s

## Data Availability

Data sharing is not applicable to this article.
